# Results of thyroidectomies according to general surgeons and otolaryngologist and cervico faciale surgeons at the general Hospital of Reference of Niamey, what differences in the protocols of management?

**DOI:** 10.1186/s12893-023-02305-y

**Published:** 2024-01-18

**Authors:** A. Saidou, A. B. Djafarou, A. A. Alfari, A. Zabeirou Oudou, K. Ide, O. G. Bakou, H. Younssa, L. James Didier, R. Sani

**Affiliations:** 1 Department of General and Digestive Surgery, Reference General Hospital, 12 674 Niamey, BP Niger; 2 Department of Otolaryngology and Cervico Facial Surgery, Reference General Hospital, 12 674 Niamey, BP Niger; 3 Department of Physical Medicine and Functional Rehabilitation, Reference General Hospital, 12 674 Niamey, BP Niger; 4 Department of General and Digestive Surgery, National Hospital, 238 Niamey, BP Niger; 5 Department of General and Digestive Surgery, Amirou Boubacar Diallo Hospital, 10146 Niamey, BP Niger; 6 Department of General and Digestive Surgery, Reference Hospital, Maradi, Niger

**Keywords:** Thyroidectomy, Recurrent laryngeal nerve, Otorhinolaryngology, General surgery, Reference general hospital

## Abstract

**Objective:**

To evaluate the surgical management of thyroid pathologies at the Reference General Hospital.

**Methods:**

This was a retro-prospective study over 4 years 6 months carried out in the departments of General and Digestive Surgery (GDS) and Otorhinolaryngology and Cervico Facial Surgery (ORL/FCS). It involved 182 patients who underwent thyroid surgery.

**Results:**

A frequency of thyroidectomy of 9.46% was found. Females predominated with a sex ratio of 0.1. The average age of patients was 42.85 years, a standard deviation 12.80. 84.06% of patients had consulted for anterior cervical mass. EU-TIRADS score 3 represented 7,14% of cases. Heteromultinodular goiter was the main indication for thyroid surgery (59.34%). Total thyroidectomy was the most commonly performed gesture in general surgery in 88,23% (*n* = 105), in Otorhinolaryngology, it was in the same proportion as lobo-isthmectomy at 47.61% (*n* = 30). The first route was video-assisted thyroidectomy 2.2% (*n* = 4). The recurrent laryngeal nerve was dissected and seen in 159 cases (87.36%) and parathyroid glands were also seen and preserved in 58.24% of cases (*n* = 106). In immediate postoperative surgery, the main complications were unilateral recurrent paralysis with dysphonia in 3.3% (*n* = 6) and compressive hematoma in 2.2% (*n* = 4). No deaths had been recorded.

**Conclusion:**

Total thyroidectomy was the most performed procedure in department of General and Digestive Surgery. Routine oral calcium and vitamin D supplementation in the general surgery ward, reduces the occurrence of hypocalcemia after total thyroidectomy and allows a safe and early exit. Standardizing protocols will further reduce complications.

## Introduction

Surgery remains an important part of the therapeutic arsenal of thyroid diseases [1]. In Niger, thyroid surgery is performed by digestive surgeons [2, 3], and Otorhinolaryngology and Facial Cervico Surgery (ORL/FCS) surgeons [4]. The success of a good thyroid intervention lies in the preservation of noble cervical structures (especially nerve and parathyroid). Identifying the recurrent laryngeal nerve (NRL) and preserving the vascularization of parathyroides, contribute to better results [5]. Postoperative hematoma or acute dyspnea are also reported as major complications, but today much rarer, they can quickly be life-threatening [6]. In Niger, the best results of thyroidectomies were obtained through surgical dissection and identification of NLR without the use of monitoring [7]. Thyroid surgery at HGR is done in two departments, with sometimes different protocols. The aim of this work is to evaluate the results of the two services in terms of complications, according to the attitudes, the indications of each and to ask the question, should we systematically supplement the patients with calcium and vitamin D after a total thyroidectomy?

## Patients and methods

It was a retrospective and prospective study over 4 years 6 months including a period of 2 years 3 months each, from January 1, 2018, to June 30, 2022. It included all patients who received thyroidectomy regardless of indication, sex or age. The results of the study came from two surgical departments: General and Digestive Surgery (GDS) and Otorhinolaryngology and Cervico Facial Surgery (ORL/FCS) of the Reference General Hospital. In both teams, the recurrent laryngeal nerve (RLN) and parathyroid glands were systematically sought through anatomical tissue dissection without nerve neurostimulation materials. For hemostasis, ligation of the arteries was done with resorbable thread 2/0, or 3/0, and also through to bipolar clamp and or thermo-fusion material.

The preparations specific to General Surgery are a premedication with Lugol drop pre-operative for 2 weeks to minimize the risk of bleeding by a vasoconstriction effect, the use of corticosteroids type Betamethasone, injected per operation and continued until J2 when it was large goiters to prevent tracheomalacia. In case of total thyroidectomy, calcium gluconate was systematically infused at J0 and continued orally at discharge. Low-dose Levothyroxine (50 micrograms) was also introduced from J2 orally to be corrected and adapted by the endocrinologist. Calcemia and thyroid control (T4, TSH) were performed only in outpatient at J3 and J21 postoperative respectively. Two post-operative consultations with the surgeon were organized before entrusting the patient to endocrinologists for hormonal follow-up.

In ORL the prescription of calcium was not systematic whatever the operating procedure, it is indicated only in case of clinical manifestations of hypocalcemia, as well as Levothyroxine.

In the case of complications such as nerve damage, the two services have in common the realization of a nasofibroscopy and a treatment by a corticosteroid therapy associated with proton pump inhibitors. In addition to this in General Surgery was associated a rehabilitation that aims to recover the voice by strengthening the phoneatory muscles and toning the vocal cords.

The chi-square test was used to look for an association between the different variables. The association was statistically significant if the *p*-value was less than or equal to 0.05. It was particularly studied the postoperative complications of the two services and compared to the operative techniques and a review of the literature.

## Results

During the study, 3072 patients including 682 in Otorhinolaryngology and Cervico Facial Surgery (ORL/FCS) and 2390 in General and Digestive Surgery (GDS) had benefited from a surgical intervention programmed any pathology, among which, 182 cases of thyroidectomy or an overall frequency of 16.87%. According to the services, this frequency was 9.23% (*n* = 63) in ORL and 4.97% (*n* = 119) in GDS. The sex ratio was 0.1, the average age of patients was 42.85 years, a standard deviation 12.80 years. There were 3.84% (*n* = 7) of patients who had a personal history of thyroid surgery and 5.5% (*n* = 10), a family history of goiter. At the initial assessment, 14.84% (*n* = 27) of patients were hyperthyroidism, 2.20% hypothyroidism (*n* = 4) and 7.70% hypocalcemia (*n* = 14).

All patients underwent an anterior cervical ultrasound, which allowed to make the diagnosis, but also to establish the EU-TIRADS classification in 27 cases (14.83%). The EU-TIRADS 3 score represented 7.14% (*n* = 13). Pre-operative cytopuncture was performed in 16 patients (8,80%) and showed a vesicular neoplasm appearance in 2.75% (*n* = 5). In the series, 34.61% (*n* = 63) of patients had received preoperative nasofibroscopy, 2 cases of vocal cord nodule (1.1%) and in one case (0,54%) glottis appreciation was difficult (large goiter).

**Fig. 1:**
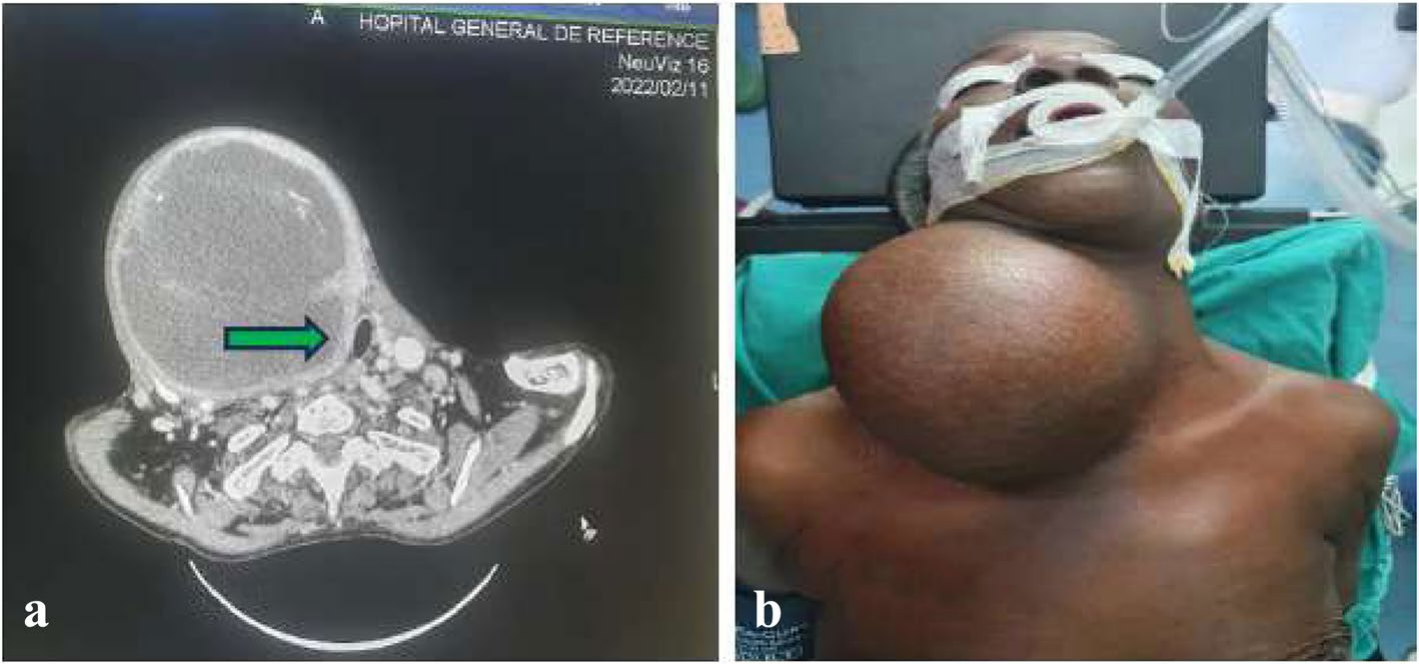
**a** Cervical-thoracic scan of anterior cervical mass (Patient A), confirming tissue-fluid mass of interest to the right thyroid lobe and compressing the trachea to the left (Green arrow). **b** Installation of patient A, neck in hyperextension, after general anesthesia and orotracheal intubation assisted by video laryngoscopy

A cervical-thoracic CT scan was performed in 5 patients (2.75%), the indication was for the large volume of goiter either looking for a plunging goiter or tracheal compression (Fig. 1a), or for suspicion of malignancy; in one case, cancer was strongly evoked. The chest radiograph showed a deviation of the trachea in 37.07% (*n* = 62) caused by thyroidian mass. In 21.98% of cases, (*n* = 40), medical treatment was performed preoperatively to stabilize hypocalcemia (Calcium and Vitamin D), hypothyroidism (levothyroxine) and hyperthyroidism (Neomercazole, betabloquant). Low Lugol premedication was done in 66.38% of patients (*n* = 79) preoperatively.

The pre-anaesthetic consultation classified 47.25% of patients (*n* = 86) ASA I. All patients were in euthyroidism prior to surgery and had received general anesthesia with orotracheal intubation (Fig. 1b), assisted by laryngoscopy video in 10.99% (*n* = 20) where pre-anaesthesia consultation had suspected difficult intubation. As a type of incision, transverse cervicotomy (Kocher type) was performed in 96.13% (*n* = 175), followed by video-assisted cervicotomy in 2.20% (*n* = 4). The bimastoidian incision was performed in 3 patients for whom bilateral functional lymph node dissection was indicated.

**Fig. 2:**
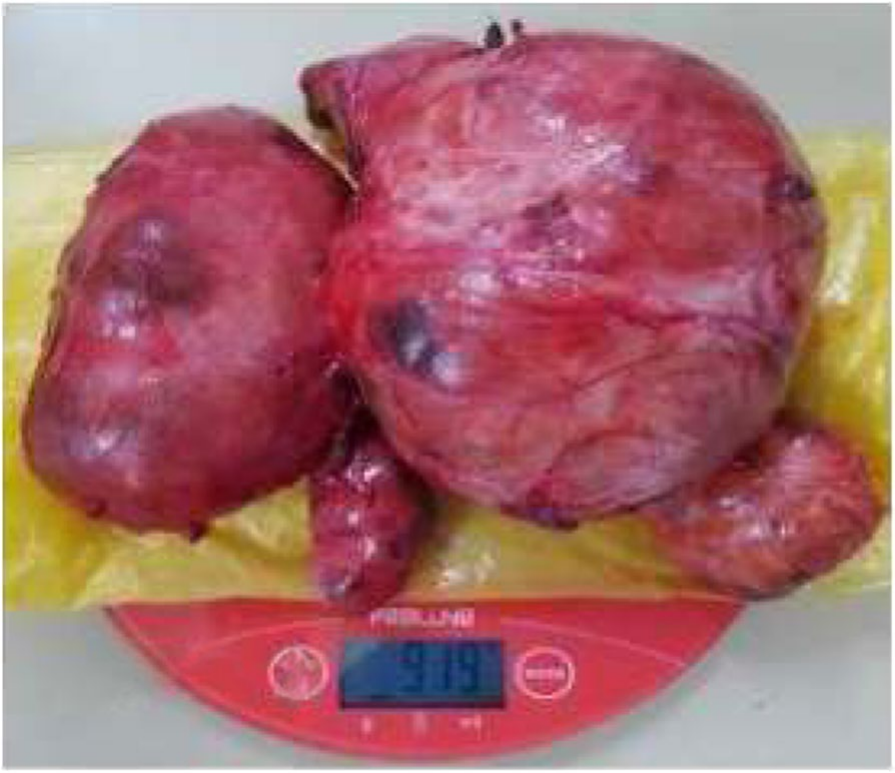
operative specimen, after total thyroidectomy for a large multinodular goiter

**Fig. 3:**
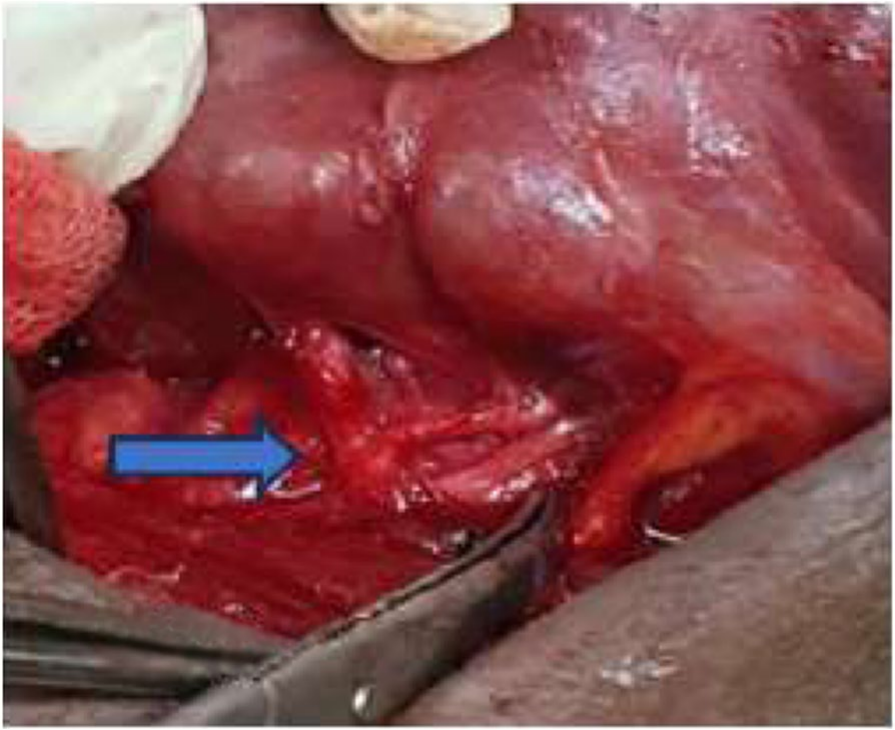
Operative identification of the RLN (scissors tip), nerve in retro position Inferior thyroid pedicle (blue arrow)

Nodular goiter was the predominant operative indication in 59.34% of cases (*n* = 108) (Table 1). Total thyroidectomy (TT) (Fig. 2) was performed in just under ¾ of the cases, 74.17% (*n* = 135). It was indicated in most cases for a multinodular goiter 52.19% (*n* = 95). According to the services, TT accounted for 88.23% of gestures in general surgery; however in ORL-FCS, lobo-isthmectomy and total thyroidectomy were performed in equal proportions (47.61% each). For the detection and identification of RLN, it was seen and preserved (Fig. 3) in 87.36% of cases (*n* = 159). Parathyroid glands were seen and preserved in 58.24% of cases (*n* = 106). In 5 cases (2.75%), a functional cervical lymph node dissection was performed, it was 4 cases of goiter with adenopathy and one case of laryngeal cancer extended to the thyroid gland. Only patients who had a video-assisted approach 3.85% (*n* = 7) were not drained.

**Table 1:** Surgical procedures according to the operative indication

Surgical procedures	Indications	Effective	Pourcentage (%)
Totale thyroidectomy	Nodular goiter	95	52,19
	Basedow	29	15,93
	Thyroid cancer	5	2,75
	Large goiter	3	1,65
	Plunging goiter	2	1,1
	Vesicular Néoplasm	1	0,55
Lobo-isthmectomy	Thyroid nodule	20	10,98
	Nodular goiter	10	5,5
	Goiter recurrence	2	1,1
	Laryngeal cancer (associated gesture)	1	0,55
Lobectomy	Thyroid nodule	4	2,2
	Goiter recurrence	4	2,2
	Large goiter	1	0,55
	Totalization for cancer	1	0,55
Subtotal thyroidectomy	Nodular goiter	3	1,65
Isthmectomy	Isthmic nodule	1	0,55
Total		**182**	**100**

In general surgery, all patients who received TT, 57.69% (*n* = 105) received supplementation with calcium gluconate and Levothyroxine.

**Fig. 4:**
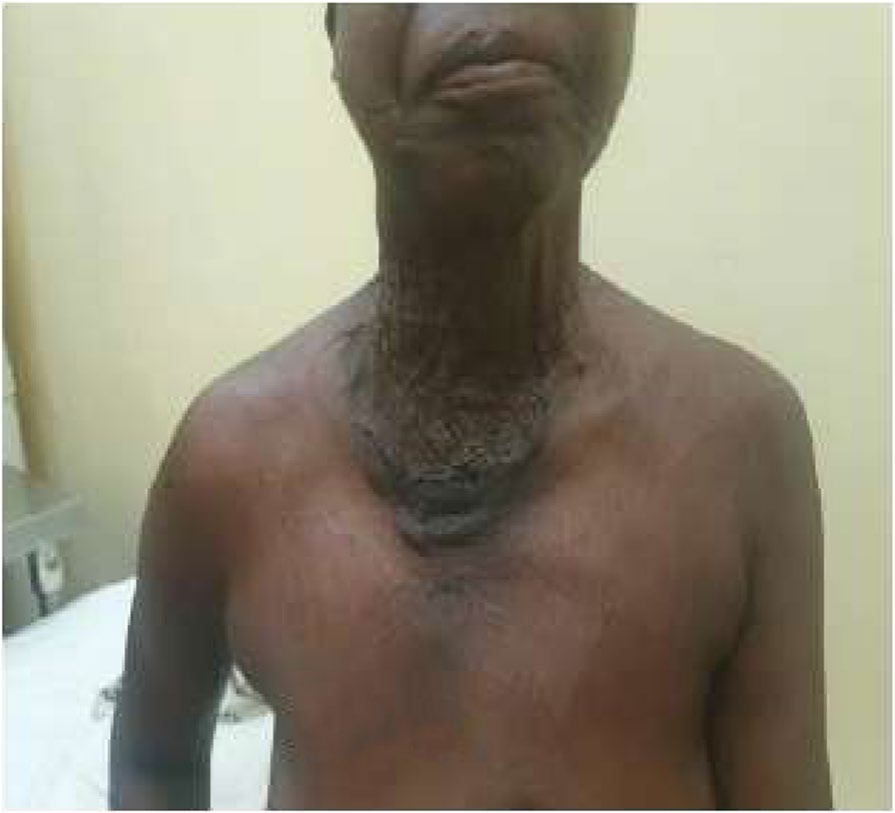
Scar, (Patient A) at J7 postoperative after puncture of the sero hematic fluid

Early postoperative complications were 7.14% (*n* = 13). It was 2.2% (*n* = 4) of compressive hematoma including 2 cases (Clavien-Dindo IIIb), 3.3% (*n* = 6) of dysphonia and 1.65% (*n* = 3) of transient hypocalcemia. Among the 6 cases of dysphonia, the operative report had not given precision in the visualization of RLN in 3 cases and in the other cases, it was visualized during dissection. In the medium term, the complications were 7.14% (*n* = 13) symptomatic transient hypocalcaemia, 3.85% (*n* = 7) subcutaneous serohematic collections (Clavien-Dindo IIIa), and 0.55% (*n* = 1) compressive hematoma (Clavien-Dindo IIIa). In the long term, it was 2.75% (*n* = 5) of definitive hypocalcaemia and 1.1% (*n* = 2) of symptomatic hypothyroidism found in the ORL series. The management of the 6 complicated dysphonia patients was done with corticosteroids. Among them, three patients had benefited in addition to physiotherapy and exercises on phonation and recovery was total. The sero-hematic collection was punctured and evacuated (Fig. 4) (Clavien-Dindo IIIa). All patients with hypocalcemia were supported by supplementation with calcium gluconate and vitamin D. In the early operative suites, 9.52 and 5.88% of complications were found in the ORL and general surgery departments respectively (Table 2), there was no statistically significant correlation between early complications and surgical specialty (*p* = 0.43). There was no hypocalcemia in the series of general surgery. Total thyroidectomy was the operative procedure that presented most of the complications including dysphonia. There was no statistically significant correlation (*P* = 0.34) between surgical intervention and early complications (Table 3).

**Table 2:** Distribution of complications by surgical specialty

Early complications	Surgical specialties	
	ORL/FCS	GDS
Dysphonia	1	5
Compressive hematoma	2	2
Symptomatic hypocalcaemia	3	0
Total	**9,52% (*n*** **= 6)**	**5,88% (*n*** **= 7)**

*P* = 0,43

**Table 3:** Distribution of early complications by surgical procedure

Early complications	Operative gestures	
	Total thyroidectomy	Lobo-Isthmectomy
Dysphonia	5	1
Compressive hematoma	2	2
Symptomatic hypocalcaemia	3	0
Total	**5,49%(*n*** **= 10)**	**1,65%(*n*** **= 3)**

*P* = 0,34

Benign pathology was the majority with 53.3% (*n* = 97), thyroid cancer represented 4.95% (*n* = 9) of which 1.65% (*n* = 3) papillary carcinoma and 3.3% vesicular carcinoma (*n* = 6). The average length of hospitalization was 2.74 days with extremes of 2 and 8 days. For patients with a video-assisted approach, the average length of hospitalization was 1.25 days. There were no deaths recorded in the series.

## Discussion

Thyroid surgery occupies a significant place in the General Hospital of Reference (HGR), it is also well developed in sub-Saharan Africa [3, 4, 8–10]. In Africa, thyroid surgery is increasingly performed by both ORL/FCS surgeons and digestive surgeons as in our structure [3, 11–14]. The training schools being different, the protocols sometimes diverge, however the main objective for the two teams is to contribute to the preservation of the integrity of the noble cervical structures (especially nervous and parathyroid) [6].

Our main indication was multinodular goiter in just over half of the cases, as reported by Vodouhé UB and al. in Benin, Leye A and al. in Senegal, and Peix JL and al in France which had found 46.7, 51.32 and 60% respectively [10, 15, 16].

The surgical approach of the thyroid through by Kocher cervicotomy was the most used in our series, it remains, even in the West the «gold standard» of thyroidectomy, reported by Peix JL, Lyon 2017. Minimally invasive surgery, increasingly used in developed countries such as thyroid video surgery, was reported in our series on a low frequency, it is also the first in Niger. This minimally invasive approach takes a growing place in the operative arsenal of benign pathologies of thyroid; it is done in well-selected patients, clearly demonstrates excellent results in terms of cure rate and patient comfort, with a shorter hospital stay, a reduction in postoperative pain and the most attractive aesthetic results [17, 18]. It is still little practiced in sub-Saharan Africa [11–13].

In general surgery, total thyroidectomy (TT) remains the most indicated technique. Our results were similar to those of Danaoui Y et al. [19] in Morocco which found 74% and Diédhiou D and al [20] 85.2% and Baldé D and al 66.92% [8] all in Senegal. On the other hand, in ORL, radical surgery is less privileged, in favor of conservative surgery as in our series and in other ORL/ FCS services [4, 12, 13]. About 10 years ago and in several studies, the most used surgical technique was subtotal thyroidectomy (STT), Xu X in China in 2005 and Thomusch O in Germany in 2003 had found respectively 63.30 and 88.16% [21, 22]. Today there is an evolution from STT, to almost total thyroidectomy and now TT. This indication of TT for benign pathology is preferred to prevent the risk of recurrence and surgical re-operation [16, 23, 24]. Nevertheless, the lobo-isthmectomy retains its indications in front of a single nodule and even finds its place for the treatment of cancers of small size and corresponding to lesions with low evolutionary risk (PEIX JL et al., Lyon 2017).

In the literature, the two major complications of immediate postoperative thyroid surgery are hypocalcaemia, whose reported incidence was between 20 to 30% [6, 25, 26] and recurrent paralysis of about 5 to 11% [6]; on the other hand bilateral recurrent involvement and or compressive hematoma, complications that can quickly put the vital prognosis in play by tracheal compression, occur at a lower frequency (less than 0.1%) according to Rosato L et al. in a multicentre study on complications of thyroid surgery in Italy in 2004 [27]. These complications are now decreasing thanks to the development of RLN identification techniques (recurrent monitoring) and the refinement of surgery (using thermo fusion equipment) that contribute to the preservation of these nerves and the vascularization of parathyroids [16]. The main risk factors for these complications according to Rosato L et al. [27] were patients (history of thyroidectomy), existing thyropathy (cancer or thyroiditis), extent of resection (lymph node dissection) and surgeon activity volume. In our series the laryngeal nerve had been dissected and visualized in 87.36% of patients, in the other cases, it had not been identified and or not sought. We perform a surgical and anatomical dissection in search of this nerve by taking as reference the lower thyroid vessels until its penetration into the larynx. However, in immediate postoperative we reported 3.3% dysphonia by partial involvement of RLN despite the high frequency of its surgical location. Its damage would certainly be related to the excessive use of bipolar electric scalpel. It is reported that visual identification of NLR remains the gold standard for nerve preservation, and according to Barczyn ski M et al., it is up to each surgeon to decide whether to use monitoring as a technique for identifying thyroid procedures or to reserve it for difficult cases [28]. Some series reported good results in nerve preservation thanks to this surgical dissection and direct visualization of the RLN [4, 7, 29]. In our study, there was no statistically significant correlation between surgical intervention and postoperative complications; As well as some authors, do not find any difference in terms of the occurrence of complications according to whether it was a TT or a TST [30–32].

The preservation of parathyroids prevents permanent hypoparathyroidism, in case of TT, although rare and their delicate manipulation allows the preservation of their vascularization [14, 33]. Does preventive supplementation with calcium and vitamin D help to avoid symptoms? It has already been shown that this systematic prevention in perioperative is effective in reducing the incidence and severity of hypocalcemia after TT, associated or not with a central lymph node dissection, without inhibiting the secretion of parathyroid hormone; and allows for safe early exit [34–37]. Better, a controlled study, randomized published in January 2023 in the International Journal of Surgery had shown that oral perioperative calcium and vitamin D supplementation significantly reduced the risk of symptomatic and biochemical hypocalcaemia compared to supplementation postoperative oral and even reduces the recovery period of symptomatic hypocalcaemia to less than 24 hours [38]. In general surgery, all patients who received TT are systematically supplemented with calcium and oral vitamin D despite the preservation of the parathyroid glands and regular monitoring was carried out during consultations (Manipulation of the parathyroid glands makes them inactive for several days, which explains the supplementation with calcium and vitamin D systhematic in the GDS). There is no hypocalcemia in this series unlike that of the ORL which did not systematically give calcium and some cases of symptomatic hypocalcemia had been found. This was reported in the ORL series of Djafarou AB and al where only symptomatic patients were treated; as the parathyroid glands were systematically researched and preserved [14]. However, the limit of this study lies in the impossibility of the dosage of parathormone in postoperative.

## Conclusion

The protocols for the surgical management of thyroid disease in the two surgical departments of the Hôpital Général de Référene are very different apart from the systematic research of recurrent laryngeal nerve and parathyroids that they keep in common. Surgical dissection in search of the recurrent nerve has shown its evidence in the preservation of nerve damage. Routine oral calcium and vitamin D supplementation in the general surgery ward, reduces the occurrence of hypocalcemia after total thyroidectomy and allows a safe and early exit. A standardization of protocols will allow a better surgical management of thyroid pathologies at the General Hospital of Reference to reduce the morbi-mortality of this surgery.

## Data Availability

No datasets were generated or analysed during the current study.

## Abbreviations

HGR: Hôpital Général de Référene
ORL/FCS: Otolaryngology and Facial Cervico Surgery.
GDS: General and Digestive Surgery
EU-TIRADS score: European-Thyroid Imaging Reporting and Data System
RLN: Recurrent laryngeal nerve
TT: Total thyroidectomy
STT: Subtotal thyroidectomy
T4: Thyroxine 4
TSH: Thyroid Stimulating Hormone
CT scan: Computed Tomography scan
ASA: American Society of Anesthesiologists
J0: Day of surgery
J2: Second postoperative day
J21: 21st post-operative day
